# Polygenic risks and cardiovascular treatment effects in severe mental illness

**DOI:** 10.1097/YPG.0000000000000401

**Published:** 2025-10-23

**Authors:** Kai Yao, Alexandra Burton, Samira Heinkel, David Osborn, Nick Bass, Andrew McQuillin

**Affiliations:** a Molecular Psychiatry Laboratory, Division of Psychiatry, Faculty of Brain Sciences, University College London; b Centre for Psychiatry and Mental Health, Wolfson Institute of Population Health; c Division of Psychiatry, Faculty of Brain Sciences, University College London, London, UK

**Keywords:** cardiovascular risk, polygenic risk, primary care, risk prediction, severe mental illness

## Abstract

**Objective:**

Patients with severe mental illness (SMI) experience increased cardiovascular risks, leading to reduced life expectancy. Polygenic risk scores (PRS) prediction is promising for assessing cardiovascular risks. This study evaluated the predictive utility of cardiovascular PRS and the impact from risk-reducing interventions among patients with SMI.

**Methods:**

Using samples from the PRIMROSE programme, involving longitudinal cardiovascular interventions within primary care, we calculated seven cardiovascular and two psychiatric (bipolar/schizophrenia) PRS to predict seven corresponding cardiovascular measures [total cholesterol/high-density lipoprotein cholesterol/low-density lipoprotein cholesterol (LDL)/triglyceride/systolic blood pressure/diastolic blood pressure/BMI] assessed at baseline and 12-month follow-up. We applied multiple linear regression models at the two time points and explored the interactions between cardiovascular and psychiatric PRS on these treatment outcomes.

**Results:**

At baseline, most cardiovascular PRS were associated with the respective measures, except LDL. At follow-up, the participants showed significant improvements in total cholesterol and systolic blood pressure measures; however, these two PRS’s prediction effects attenuated toward the null. LDL measures became negatively associated with bipolar PRS posttreatment, though no significant interaction effects were found. Participants in the highest bipolar PRS quartile group had 0.58 mmol/L lower LDL measures than the lowest quartile group at follow-up. These results were robust to potential power reduction, participants’ age, sex, prescribed medications, smoking habits, alcohol consumption, and physical activity.

**Conclusion:**

Our findings underscore the dynamic interplay between genetic risks and treatment effects on cardiovascular outcomes in SMI and warrant careful PRS assessment timing. While the clinical utility of PRS is still evolving, future research should explore different disorders’ subtype-specific genetic interactions with interventions.

## Introduction

Patients diagnosed with bipolar disorder, schizophrenia (SCZ), or psychosis are often jointly referred to as patients with severe mental illnesses (SMI). Patients with SMI have a higher mortality rate, leading to a 15–20 year reduction in life expectancy in comparison to the general population (Nordentoft *et al.*, 2013). Cardiovascular disease (CVD) contributes 17.4 and 22.0% of life years lost in males and females with SMI, respectively (Jayatilleke *et al.*, 2017; Nielsen *et al*., 2021). CVD was recorded as the cause of death for approximately 24% of patients with SCZ and 38% of patients with bipolar disorder in the European population (Nielsen *et al.*, 2013; Westman *et al.*, 2013). The life expectancy gap between the general population and patients with SMI appears to be widening because of the lack of effective interventions (Hayes *et al.*, 2017).

Multiple factors underlie the increased risks of cardiovascular events among patients with SMI (Nielsen *et al.*, 2021). Patients with SMI are more likely to have an unhealthy lifestyle, increased sedentary time, lack of exercise, poor diet, and increased rates of smoking and heavy alcohol use (Hartz *et al.*, 2014; Vancampfort *et al.*, 2015, 2016, 2017). Treatments with antipsychotic drugs can lead to weight gain and higher cardiovascular mortality (Rotella *et al.*, 2020). In addition, antidepressants and mood stabilizers can cause adverse metabolic effects (Correll *et al.*, 2015; Pérez-Piñar *et al.*, 2016).

People with SMI may also have a higher genetic predisposition to CVD compared with the general population (O’Sullivan *et al.*, 2022). A meta-analysis of genome-wide association studies (GWAS) and candidate gene studies identified 24 potential pleiotropic genes that are likely to be shared between mood disorders and cardiometabolic disease risk (Amare *et al.*, 2017). Patients with SCZ were shown to have distinct metabolic profiles according to multidimensional scaling on shared genetic variants between psychiatric and cardiometabolic disorders (Strawbridge *et al.*, 2021). In addition, researchers compared polygenic risk scores (PRS) for SCZ and bipolar disorder, which summarize the genetic predispositions to these two disorders, with 28 additional cardiometabolic traits (So *et al.*, 2019). The results showed that SCZ PRS was associated with several cardiometabolic abnormalities, including glucose metabolism abnormalities and adverse adipokine profiles, independent of medication use. In contrast, bipolar disorder PRS showed associations with an overall more favorable cardiometabolic profile, suggesting potential protective metabolic traits linked to bipolar disorder genetic risks; thus, cardiometabolic abnormalities in bipolar disorder are more likely to be secondary.

Various behavioral interventions have been developed to address cardiovascular risks for patients with SMI (Goldfarb *et al.*, 2022). Several of these developed interventions have been found to be effective in controlling cardiovascular risks among people with SMI (Jakobsen *et al.*, 2017; Aschbrenner *et al.*, 2022). The added value from incorporating cardiovascular PRS in cardiovascular risk predictions is increasingly recognized (Sun *et al.*, 2021; Samani *et al.*, 2024). Individuals with higher cardiovascular genetic risks were found to benefit more from statin and alirocumab treatment, suggesting potential clinical utility of PRS assessments (Natarajan *et al.*, 2017; Damask *et al.*, 2020); however, genomic and clinical risk factor predictions for CVD may vary over the life course (Urbut *et al.*, 2025). In addition, no previous studies have specifically examined how interventions or treatments can influence PRS-based predictions of cardiovascular risks in patients with SMI over time. Meanwhile, the impact of psychiatric genetic risk on cardiovascular treatment effectiveness remains unclear.

The PRIMROSE group developed a pragmatic intervention aimed at reducing CVD risk factors among people with SMI in primary care in England (Burton *et al.*, 2015). The PRIMROSE intervention was tested in a clinical trial, and a similar effect was observed on total cholesterol concentration reduction at 12 months as treatment-as-usual groups, with significantly decreased costs through decreased psychiatric relapses and hospital readmissions in the treatment arm (Osborn *et al.*, 2018). A subset of the trial participants provided consent for genetic data analysis to determine whether their genetic profiles can predict variations in treatment response.

The overall aim of this study was to use seven cardiovascular and two psychiatric (bipolar disorder/SCZ) PRS to understand how genetic risks reflect cardiovascular treatment effectiveness. The seven cardiovascular measures included total cholesterol, high-density lipoprotein cholesterol (HDL), low-density lipoprotein cholesterol (LDL), triglyceride, systolic blood pressure, diastolic blood pressure, and BMI. Specifically, we wanted to explore if these PRS predictions would change after treatments and how psychiatric genetic risks modify these predictions. We hypothesized that the cardiovascular PRS would interact with psychiatric PRS (bipolar disorder/SCZ) in predicting treatment effectiveness. People with higher genetic risks for both cardiovascular risks and psychiatric disorders were expected to have reduced treatment response, given this group of patients’ potential adverse metabolic profiles.

## Methods

### Participants selection

Participants were recruited from general practitioner practices across both rural and urban areas of England (Osborn *et al.*, 2018). Ethics approval was obtained from the City Road and Hampstead Research Ethics Committee (Reference No: 12/LO/1934, approval granted 10 January 2013). Local National Health Service approvals were obtained before the start of each recruitment wave. At baseline, the recruited participants were randomized into a PRIMROSE intervention group, which involved regular appointments with a practice nurse to support cardiovascular health behavior change over a 6-month period or a treatment-as-usual group where the participants received regular care (Osborn *et al.*, 2018). In the original study, the PRIMROSE intervention was more cost-effective than usual treatment, with no significant differences found in participants’ treatment response between the two treatment groups. A subset of the participants (*N* = 194/326) consented to provide a saliva sample for genotyping and the conduct of genetic research.

### Measures

The participants’ total cholesterol, HDL and LDL, triglycerides, blood pressures (systolic and diastolic), and BMI were included for analyses. These measures were collected at baseline using practice equipment and procedures and repeatedly taken at 12-month follow-up following the same procedure. We excluded outliers beyond 3 SDs from the mean in the raw clinical measurements at both time points to minimize potential bias from extreme values. Other health and lifestyle measures included medication use in the past 12 months (antipsychotic/antidepressant/mood stabilizer/antihypertensive/other medications); smoking habits (number of cigarettes per day); alcohol use [Alcohol Use Disorders Identification Test Score (AUDIT-C)]; and physical activity measures using the International Physical Activity Questionnaire (IPAQ). These data were collected either directly from participant interviews, clinical measures, or from the practice medical records (Osborn *et al.*, 2018).

### Genetic data imputation and quality control

A total of 194 participants’ genetic data were genotyped by UCL Genomics on the Global Screening Array then received stringent quality control (Anderson *et al.*, 2010). The process focused on excluding samples with discordant sex, missing genotype data above 10%, excessive heterozygosity (more than 3 SDs above the mean), and evidence of relatedness [proportion of IBD (identity by descent) shared (PIHAT) >0.2]. Single-nucleotide polymorphisms (SNPs) that deviated substantially from the Hardy–Weinberg equilibrium (*P* < 10^−6^) were excluded. We also excluded SNPs with a minor allele frequency less than 0.5% to maintain broad reference coverage while removing extremely rare variants, which could be prone to artifacts. All of these described quality control steps were performed using PLINK 1.9 (Chang *et al.*, 2015).

Imputation was performed using the Sanger Imputation server. Before the upload, the data genotypes were prepared as instructed, and checks were performed using the HRC-1000G-check-bim tool Version 4.2.3 (Rayner 2015). SNPs were uploaded for imputation, applying the Haplotype Reference Consortium reference panel (release 1.1) (McCarthy *et al.*, 2016). Prephasing was done with EAGLE v.2.3.3, and imputation was done with positional Burrows–Wheeler transform (Durbin, 2014). Postimputation quality control repeated the prior quality control procedure and further excluded all SNPs with info less than 0.9.

### Polygenic risk scores calculations

Patients’ cardiovascular and psychiatric PRS were computed from the imputed genetic data with the Polygenic Risk Score-Continuous Shrinkage (PRS-CS-auto) method, which employs the Bayesian theorem for a continuous shrinkage to provide a single score for each sample (Ge *et al.*, 2019). The PRS-CS-auto method was chosen over other methods because it outperformed other existing methods according to the simulation studies (Ge *et al.*, 2019; Pain *et al.*, 2021) and has been applied to the predictions of various complex traits (Grigoroiu-Serbanescu *et al.*, 2020; Yao *et al.*, 2023).

The application of the PRS-CS method required a linkage disequilibrium reference panel and reference GWAS summary statistics, which are used to infer the posterior effect sizes of SNPs. We chose the European sample from The 1000 Genomes Project Consortium (2010) as our linkage disequilibrium reference panel. We used GWAS summary statistics on European samples from the Global Lipids Genetics Consortium for total cholesterol, HDL cholesterol, LDL cholesterol, and triglycerides (Graham *et al.*, 2021). We used the latest available GWAS summary statistics on European samples for blood pressure (diastolic/systolic) (Keaton *et al.*, 2024), BMI (Yengo *et al.*, 2018), bipolar disorder (O’Connell *et al.*, 2025), and SCZ (Trubetskoy *et al.*, 2022).

For the lipid traits, variances explained by the index variants in the European population were estimated as 11% for total cholesterol, 10% for HDL, 12% for LDL, and 9% for triglycerides (Graham *et al.*, 2021). The authors also applied PRS-CS to calculate LDL PRS for LDL measures as an estimation and found that it could explain 18.2% of the variance in measured LDL from the European population. For the two blood pressures, the authors applied SBayesRC, an alternative Bayesian method for PRS scoring (Keaton *et al.*, 2024). The PRS were estimated to explain 12.12% of the variance in diastolic and 11.37% of the variance in systolic blood pressure, respectively. For BMI, the authors applied the Conditional and Joint analysis method for an estimation at a threshold of 0.001 (Keaton *et al.*, 2024). The selected SNP could explain approximately 13.9% of the variance in sample BMI with a corresponding prediction *R*^2^ of approximately 10.2%. For bipolar disorder, PRS calculated from PRS-CS was estimated to explain a median of approximately 7.4% variance pooling from all European cohorts (O’Connell *et al.*, 2025). For SCZ, the authors conducted leave-one-sample out PRS analyses using different *P* thresholds for PRS constructions (Trubetskoy *et al.*, 2022). At a 0.05 threshold, which maximizes out-of-sample prediction, the median variance in PRS liability explained was found to be 7.3%.

### Statistical analysis

The participants’ summary statistics were first presented in respect to their treatment group for all demographic and clinical variables. To replicate prior findings and evaluate potential subset-specific effects, we conducted *t* and *χ*^2^ tests based on the variable type for both baseline and 12-month follow-up measures, comparing the two groups. We conducted multiple linear regressions taking the seven cardiovascular measures at baseline as outcome measures and each of their associated PRS as predictors for associations before treatment. We conducted the same set of analyses on cardiovascular measures at 12-month follow-up to explore how treatments can influence PRS predictions. To address potential power reduction from participant dropout at follow-up, we calculated and compared the power of each pair regression models using Cohen’s *f*^2^ method at 0.05 significance level as a sensitivity check (Cohen, 2013). We then explored the interactions between cardiovascular and psychiatric PRS on the 12-month follow-up measures to test how psychiatric genetic risks can interfere with cardiovascular treatment effectiveness. In addition, we split the bipolar disorder PRS into four quartile groups for further analyses and conducted subgroup analyses for the two treatment groups as sensitivity analyses. Sensitivity analyses on only European participants were also conducted.

Covariates in all analyses included the participants’ age, sex, treatment allocation, diagnosis, smoking habits, alcohol consumption, physical activity measures, and 10 principal components from the genetic data for population structure. Each model was also adjusted for the corresponding medication presence recorded at baseline or 12-month assessment, including antipsychotic, antidepressant, antihypertensive, mood stabilizers, and other medications in binary format. Each of the 12-month analyses also included the corresponding baseline measure as an additional covariate to assess the independence of the genetic prediction. These were referred to as 12-month baseline adjustment models. Hypotheses and assumptions for each regression were prechecked and found to be satisfactory.

For multiple testing correction, we used the false discovery rate (FDR) correction methods (Benjamini and Hochberg, 1995). The FDR method was chosen over the others as it gives a good illustration of results and has been applied in previous studies involving PRS (Grigoroiu-Serbanescu *et al.*, 2020; Yao *et al.*, 2023). The correction applies a monotonic adjustment, which could result in making multiple tests having the same *P* values after adjustments, especially if in the nonsignificant range. The *P*-value threshold was set at 0.05 for adjusted *P* values. All reported analyses were conducted using RStudio with R 4.3.2 (R Core Team, 2021).

## Results

### Sample demographics

Overall, 188 participants’ genetic data passed quality control and had complete datasets at baseline. A total of 92 (49%) of them received the PRIMROSE intervention, while the rest received treatment as usual, which was taken as the control group (Table 1). The whole sample was reasonably balanced in sex (47% males), while the control group had slightly higher ratios of males (53 vs. 40%); however, there was no significant evidence to suggest the imbalance (*P* = 0.104). The overall sample contained mostly European (White) ethnicity (95%) participants, and about half of them had diagnoses of bipolar disorder (52%) and were non/ex-smokers (53%). The overall mean AUDIT-C score of 3.52 (SD: 3.47) suggested that the participants were generally at a low risk for hazardous drinking or alcohol use disorder. The overall IPAQ total score at 2133 suggested that the participants on average, meet recommended activity levels for health; however, the high SD (2249) indicated that the physical activity time varied largely between individuals. The two groups of participants did not differ in ethnicity, diagnoses, smoking/drinking habits, physical activity time, or medication use (Table 1). The PRIMROSE intervention group’s systolic blood pressure was slightly lower than the control group at the baseline (125.32 vs. 130.81; *P* = 0.027). The two groups of participants did not differ in any other clinical measures, including total cholesterol, HDL, LDL, triglycerides, diastolic blood pressure, and BMI.

**Table 1:** Participant demographics and clinical characteristics by treatment groups at baseline assessment

	*N*	Overall	PRIMROSE	Control	*P* values
Overall samples		188		*n* = 92 (49%)	*n* = 96 (51%)	
Age at assessment	188	50.21 (10.22)	50.18 (10.31)	50.23 (10.19)	0.976^a^
Sex	188				0.104^b^
Male		88 (47%)	37 (40%)	51 (53%)	
Female		100 (53%)	55 (60%)	45 (47%)	
Ethnicity	188				0.693^b^
European (White)		178 (95%)	86 (93%)	92 (96%)	
Other		10 (5%)	6 (7%)	4 (4%)	
Diagnosis	188				0.263^b^
Bipolar		97 (52%)	46 (50%)	51 (53%)	
Schizophrenia		44 (23%)	26 (28%)	18 (19%)	
Other diagnoses		47 (25%)	20 (22%)	27 (28%)	
Smoking	188				0.361^b^
Heavy (≥20 a day)		42 (22%)	24 (26%)	18 (19%)	
Moderate (10–19 a day)		28 (15%)	10 (11%)	18 (19%)	
Light (≤9 a day)		19 (10%)	10 (11%)	9 (9%)	
Non/ex-smoker		99 (53%)	48 (52%)	51 (53%)	
AUDIT-C score	188	3.52 (3.47)	3.52 (3.41)	3.51 (3.55)	0.982^a^
IPAQ total score	188	2133 (2249)	2059 (2273)	2203 (2236)	0.663^a^
Medications	188				
Antipsychotic		117 (62%)	54 (59%)	63 (66%)	0.407^b^
Antidepressant		90 (48%)	45 (49%)	45 (47%)	0.894^b^
Mood stabilizer		55 (29%)	25 (27%)	30 (31%)	0.650^b^
Antihypertensive		40 (21%)	17 (18%)	23 (24%)	0.460^b^
Other medications		162 (86%)	77 (84%)	85 (89%)	0.453^b^
Clinical and blood tests						
Total cholesterol	187	5.71 (0.85)	5.73 (0.85)	5.68 (0.86)	0.685^a^
HDL	185	1.28 (0.40)	1.30 (0.38)	1.25 (0.41)	0.405^a^
LDL	120	3.43 (0.81)	3.49 (0.84)	3.37 (0.77)	0.417^a^
Triglycerides	127	2.21 (1.22)	2.14 (1.23)	2.27 (1.21)	0.537^a^
Systolic blood pressure	188	128.12 (17.16)	125.32 (14.74)	130.81 (18.88)	**0.027** ^a^
Diastolic blood pressure	186	81.68 (10.34)	81.20 (10.26)	82.15 (10.46)	0.531^a^
BMI	185	31.60 (5.35)	31.86 (5.86)	31.34 (4.82)	0.510^a^

In bold *P* met the significance threshold at 0.05.

Other diagnoses included schizoaffective disorder, persistent delusional disorder, and other psychoses.

AUDIT-C, Alcohol Use Disorders Identification Test Score; HDL, high-density lipoprotein cholesterol; IPAQ, International Physical Activity Questionnaire; LDL, low-density lipoprotein cholesterol.

a Two-sample *t* test; mean (SD).

b Pearson’s *χ*^2^ test of independence; *n* (%).

At the 12-month follow-up, only 158 participants were available for analysis, and 77 (49%) of them had the PRIMROSE intervention. We did not observe any significant difference in any of these selected measures between these two groups as to the original study (Table 2). Comparing these measures at the two time points, the participants’ overall AUDIT-C score dropped from 3.52 to 3.08, and their IPAQ total score increased from 2133 to 3694 (Tables 1 and 2). These score changes indicated that the participants had positive improvements in some of their life habits. Overall, the interventions were effective in reducing total cholesterol measures (*t* = 2.277, *P* = 0.024). The participants’ overall systolic blood pressure dropped from 128.12 to 124.36 mmHg and their diastolic blood pressure also dropped from 81.68 to 79.68 mmHg (Tables 1 and 2). These reductions were statistically significant for systolic blood pressure (*t* = 2.243, *P* = 0.026) and close for diastolic blood pressure (*t* = 1.828, *P* = 0.068). These changes further indicated the benefits from both interventions; however, changes in all other measures were small overall, considering both interventions. The overall patterns of treatment response in our analyses are largely consistent with those reported in the original PRIMROSE study, which was expected given that we have used a subset of the original cohort (Osborn *et al.*, 2018).

**Table 2:** Participant demographics and clinical characteristics by treatment groups at 12-month follow-up

	*N*		Overall	PRIMROSE		Control		*P* values
Overall samples		158			*n* = 77 (49%)		*n* = 81 (51%)	
Age at assessment	158		51.30 (9.97)	51.21 (10.05)		51.40 (9.95)		0.907^a^
Sex	158							0.321^b^
Male			71 (45%)	31 (40%)		40 (49%)		
Female			87 (55%)	46 (60%)		41 (51%)		
Ethnicity	158							0.999^b^
European (White)			150 (95%)	73 (95%)		77 (95%)		
Other			8 (5%)	4 (5%)		4 (5%)		
Diagnosis	158							0.435^b^
Bipolar			85 (54%)	41 (53%)		44 (54%)		
Schizophrenia			35 (22%)	20 (26%)		15 (19%)		
Other diagnoses			38 (24%)	16 (21%)		22 (27%)		
Smoking	158							0.688^b^
Heavy (≥20 a day)			28 (18%)	16 (21%)		12 (15%)		
Moderate (10–19 a day)			25 (16%)	11 (14%)		14 (17%)		
Light (≤9 a day)			15 (9%)	6 (8%)		9 (11%)		
Non/ex-smoker			90 (57%)	44 (57%)		46 (57%)		
AUDIT-C score	158		3.08 (3.34)	2.82 (3.28)		3.33 (3.39)		0.333^a^
IPAQ total score	158		3694 (5218)	3621 (4515)		3764 (5836)		0.863^a^
Medications	158							
Antipsychotic			94 (59%)	47 (61%)		47 (58%)		0.823^b^
Antidepressant			81 (51%)	40 (52%)		41 (51%)		0.994^b^
Mood stabilizer			40 (25%)	17 (22%)		23 (28%)		0.466^b^
Antihypertensive			48 (30%)	21 (27%)		27 (33%)		0.513^b^
Other			144 (91%)	67 (87%)		77 (95%)		0.134^b^
Clinical and blood tests								
Total cholesterol	158		5.46 (1.11)	5.47 (1.11)		5.45 (1.13)		0.888^a^
HDL	156		1.28 (0.42)	1.28 (0.42)		1.29 (0.42)		0.919^a^
LDL	94		3.27 (0.98)	3.25 (0.91)		3.29 (1.05)		0.840^a^
Triglycerides	102		2.20 (1.26)	2.11 (1.38)		2.28 (1.14)		0.502^a^
Systolic blood pressure	156		124.36 (13.95)	122.78 (13.52)		125.86 (14.28)		0.168^a^
Diastolic blood pressure	157		79.68 (9.90)	80.01 (9.42)		79.36 (10.39)		0.679^a^
BMI	156		31.77 (5.77)	31.94 (6.26)		31.60 (5.30)		0.709^a^

Other diagnoses included schizoaffective disorder, persistent delusional disorder, and other psychoses.

AUDIT-C, Alcohol Use Disorders Identification Test Score; HDL, high-density lipoprotein cholesterol; IPAQ, International Physical Activity Questionnaire; LDL, low-density lipoprotein cholesterol.

a Two-sample *t* test; mean (SD).

b Pearson’s *χ*^2^ test of independence; *n* (%).

### Cardiovascular polygenic risk scores associations results

The PRIMROSE and treatment-as-usual groups were combined for the following analyses, with the group allocation as an additional covariate given that we did not find evidence for significant differences in their treatment effects after 12 months. The following analyses focused on exploring the participants’ treatment responses across the combined sample.

According to our baseline models, most cardiovascular PRSs were predictive of the actual cardiovascular measures among participants with SMI before any treatments except for LDL (Table 3). Most of the associations remained robust after interventions with only small attenuations in association coefficients (Table 3). After adjustment of the corresponding baseline measures, only HDL PRS remained predictive of the actual HDL measures regardless of the treatment. This might suggest that the interventions had little impact on HDL or HDL is more genetically modified among patients with SMI.

**Table 3:** Adjusted results of multiple regressions with corresponding cardiovascular polygenic risk scores

Variables	Coefficient	SE	*t* Statistics	95% CI	*P* value	*P*_adj_
Total cholesterol						
Baseline	0.196	0.063	3.116	0.072–0.319	**0.002**	**0.008**
12 Months	0.155	0.094	1.643	−0.032 to 0.342	0.103	0.166
12-Month BA	0.001	0.091	0.011	−0.179 to 0.181	0.991	0.991
HDL						
Baseline	0.166	0.024	6.822	0.118–0.214	**<0.001**	**<0.001**
12 Months	0.198	0.030	6.604	0.139–0.257	**<0.001**	**<0.001**
12-Month BA	0.056	0.025	2.257	0.007–0.105	**0.026**	**0.049**
LDL						
Baseline	0.084	0.077	1.083	−0.07 to 0.237	0.281	0.367
12 Months	0.119	0.117	1.022	−0.114 to 0.352	0.311	0.367
12-Month BA	0.037	0.118	0.314	−0.199 to 0.273	0.754	0.792
Triglycerides						
Baseline	0.440	0.139	3.171	0.165–0.715	**0.002**	**0.008**
12 Months	0.434	0.163	2.657	0.109–0.759	**0.010**	**0.025**
12-Month BA	0.253	0.175	1.443	−0.098 to 0.604	0.154	0.216
Systolic blood pressure						
Baseline	4.269	1.339	3.187	1.624–6.914	**0.002**	**0.008**
12 Months	2.093	1.185	1.765	−0.252 to 4.438	0.080	0.140
12-Month BA	1.099	1.089	1.009	−1.055 to 3.253	0.315	0.367
Diastolic blood pressure						
Baseline	1.924	0.811	2.373	0.322–3.525	**0.019**	**0.044**
12 Months	1.890	0.829	2.280	0.25–3.53	**0.024**	**0.049**
12-Month BA	1.126	0.745	1.513	−0.347 to 2.599	0.133	0.199
BMI						
Baseline	1.451	0.416	3.486	0.629–2.273	**0.001**	**0.004**
12 Months	1.376	0.500	2.752	0.387–2.365	**0.007**	**0.020**
12-Month BA	0.092	0.243	0.379	−0.389 to 0.573	0.705	0.780

*P*_adj_ are *P* values corrected using false discovery rate method. *P* values in bold are smaller than the threshold at 0.05.

Presented results are outputs from linear regressions with corresponding cardiovascular polygenic risk scores regressed on each measure. Participants’ sex, age, treatment allocation, diagnosis, smoking scores, alcohol scores, IPAQ scores, and the first 10 principal components from population stratification were added as covariates to all models. Each model was also adjusted for the corresponding medication recorded at baseline or 12-month assessment, including antipsychotic, antidepressant, antihypertensive, mood stabilizers, and other medications. 12-Month baseline adjusted models were further adjusted for the corresponding baseline measure.

BA, baseline adjusted; CI, confidence interval; HDL, high-density lipoprotein cholesterol; IPAQ, International Physical Activity Questionnaire; PRS, polygenic risk score.

**Fig. 1:**
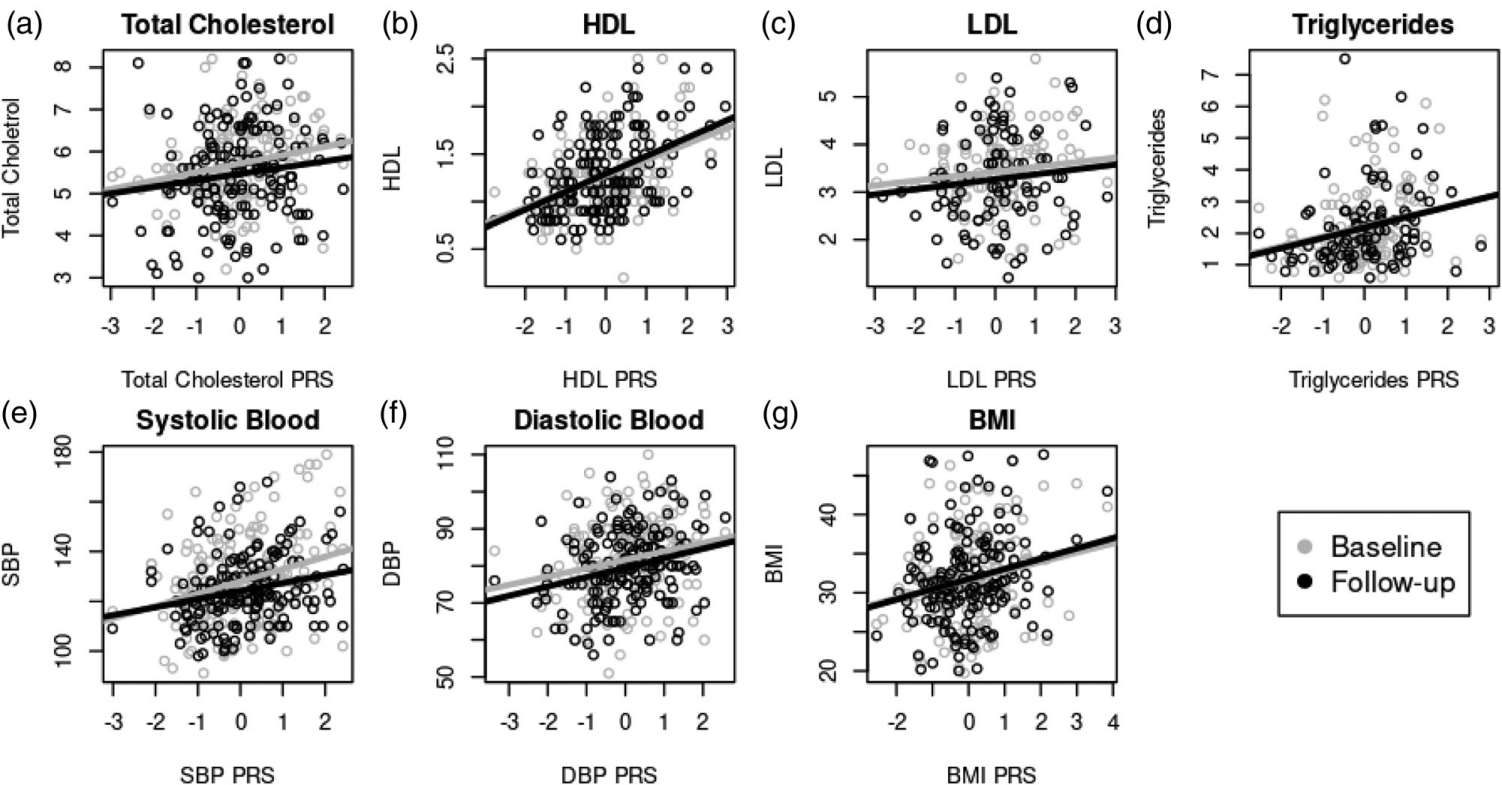
By-group scatter plots comparing baseline and 12-month follow-up cardiovascular measures against associated PRS. Fitted lines came from corresponding linear regressions, statistics reported in Table 3 for baseline and 12-month models. (a–g) Scatter plots with fitted regression lines at the two timepoints for total cholestrol, HDL, LDL, triglycerides, systolic blood pressure, diastolic blood pressure, and BMI. DBP, diastolic blood pressure; HDL, high-density lipoprotein cholesterol; LDL, low-density lipoprotein cholesterol; PRS, polygenic risk scores; SBP, systolic blood pressure.

The interventions were most effective in reducing total cholesterol (*t* = 2.277, *P* = 0.024) and systolic blood pressure (*t* = 2.243, *P* = 0.026) measures. The longitudinal comparison revealed a clear attenuation of genetic associations after effective treatment, with the predictive effects of total cholesterol and systolic blood pressure PRS weakening toward the null at the 12-month follow-up. This trend was visually evident in Fig. 1 (panels a and e), where the fitted regression lines became notably flatter compared with the baseline. In contrast, measures with minimal changes maintained stable associations, as demonstrated by overlapping or near-parallel fitted lines in Fig. 1 (panels b–d, f, and g).

The association coefficients for total cholesterol PRS dropped from 0.196 at baseline to 0.155 at 12-month follow-up, and the association coefficients for systolic blood pressure PRS dropped from 4.269 at baseline to 2.093 at 12-month follow-up (Table 3). Formal comparison of the coefficients between the two time points using *Z*-score tests revealed no statistically significant differences for total cholesterol (Δ = 0.041, SE = 0.113, *Z* = 0.362, *P* = 0.717) or systolic blood pressure (Δ = 2.176, SE = 1.788, *Z* = 1.217, *P* = 0.224); however, even these modest attenuations were sufficient to substantially weaken PRS predictive accuracy following effective treatment interventions (Table 3).

Our sensitivity analyses comparing the baseline and 12-month regression models revealed no significant difference in model power. Therefore, the observed attenuations were more likely because of changes in participants’ actual measures. In addition, our sensitivity analyses separating the PRIMROSE intervention and control group did not produce any major change in the result patterns. Sensitivity analyses on only European participants resulted in only minor changes, with the main patterns of associations maintained consistently (Supplementary Tables 1 and 2, Supplemental digital content, https://links.lww.com/PG/A330).

### Bipolar disorder and schizophrenia polygenic risk scores interaction results

We conducted multiple linear regressions taking interactions between the two psychiatric PRS (bipolar disorder/SCZ) and each cardiovascular PRS on the corresponding 12-month follow-up measures to explore the impact from psychiatric genetic risks. We first examined potential collinearity between the two psychiatric PRS and each cardiovascular PRS. We only found weak associations between SCZ PRS and BMI PRS (coefficient = −0.171; *t* = −2.247, *P* = 0.026); however, the model’s variables only had the highest variance inflation factor number of 2.414, suggesting no exceptional collinearity.

From the models, we did not find any evidence to suggest that there was any significant interaction effect between bipolar disorder or SCZ PRS and the corresponding cardiovascular PRS on these clinical measures (Table 4). At 12-month follow-up, total cholesterol PRS was no longer predictive of the actual total cholesterol measure (*P*_adj_ = 0.166; Table 3); however, for each unit increase in bipolar disorder PRS, there was a decrease in total cholesterol of 0.276 mmol/L (*P*_adj_ = 0.043). In addition, LDL PRS was not predictive of the actual LDL levels at both time points; however, for each unit increase in bipolar disorder PRS, LDL decreased by 0.331 mmol/L (*P*_adj_ = 0.043) at the 12-month follow-up, suggesting a potential protective effect from higher bipolar disorder genetic risks.

**Table 4:** Adjusted results of cardiovascular and psychiatric polygenic risk scores interactions

Variables	Coefficient	SE	*t* Statistics	95% CI	*P* value	*P*_adj_
Total cholesterol						
BD PRS	−0.276	0.091	−3.016	−0.457 to −0.095	**0.003**	**0.043**
BD PRS Interaction	0.138	0.097	1.422	−0.054 to 0.331	0.157	0.670
SCZ PRS	−0.210	0.099	−2.117	−0.407 to −0.014	0.036	0.254
SCZ PRS Interaction	0.068	0.098	0.693	−0.126 to 0.262	0.490	0.940
HDL						
BD PRS	0.004	0.030	0.139	−0.055 to 0.063	0.889	0.940
BD PRS Interaction	0.012	0.035	−0.352	−0.082 to 0.057	0.726	0.940
SCZ PRS	−0.033	0.032	−1.028	−0.096 to 0.03	0.306	0.804
SCZ PRS Interaction	−0.003	0.036	−0.075	−0.074 to 0.069	0.940	0.940
LDL						
BD PRS	−0.331	0.104	−3.195	−0.539 to −0.124	**0.002**	**0.043**
BD PRS Interaction	0.106	0.105	1.011	−0.103 to 0.315	0.316	0.804
SCZ PRS	−0.212	0.122	−1.738	−0.456 to 0.032	0.087	0.487
SCZ PRS Interaction	0.038	0.114	0.335	−0.189 to 0.266	0.739	0.940
Triglycerides						
BD PRS	−0.190	0.136	−1.395	−0.461 to 0.081	0.167	0.670
BD PRS Interaction	−0.093	0.156	−0.597	−0.405 to 0.218	0.553	0.940
SCZ PRS	−0.161	0.149	−1.082	−0.457 to 0.135	0.283	0.804
SCZ PRS Interaction	0.032	0.161	0.199	−0.29 to 0.354	0.842	0.940
Systolic blood pressure						
BD PRS	1.189	1.086	1.094	−0.961 to 3.338	0.276	0.804
BD PRS Interaction	−2.306	1.065	−2.165	−4.414 to −0.198	0.032	0.254
SCZ PRS	−0.536	1.263	−0.424	−3.035 to 1.963	0.672	0.940
SCZ PRS Interaction	0.164	0.979	0.168	−1.772 to 0.101	0.867	0.940
Diastolic blood pressure						
BD PRS	−0.327	0.830	−0.394	−1.969 to 1.314	0.694	0.940
BD PRS Interaction	−0.496	0.818	−0.607	−2.114 to 1.122	0.545	0.940
SCZ PRS	−0.603	0.925	−0.651	−2.433 to 1.228	0.516	0.940
SCZ PRS Interaction	−0.194	0.811	−0.239	−1.799 to 1.411	0.812	0.940
BMI						
BD PRS	0.319	0.466	0.684	−0.603 to 1.241	0.495	0.940
BD PRS Interaction	−0.049	0.505	−0.097	−1.048 to 0.95	0.923	0.940
SCZ PRS	−0.063	0.520	−0.121	−1.091 to 0.965	0.904	0.940
SCZ PRS Interaction	−0.057	0.460	−0.124	−0.968 to 0.853	0.901	0.940

*P*_adj_ are *P* values corrected using the false discovery rate method. *P* values in bold are smaller than the threshold at 0.05.

Presented results are outputs from linear regressions with corresponding cardiovascular polygenic risk scores and their interactions with BD or SCZ PRS regressed on each measure. Adjustments are the same as regressions described in Table 3.

BD, bipolar disorder; CI, confidence interval; HDL, high-density lipoprotein cholesterol; LDL, low-density lipoprotein cholesterol; PRS, polygenic risk scores; SCZ, schizophrenia.

**Fig. 2:**
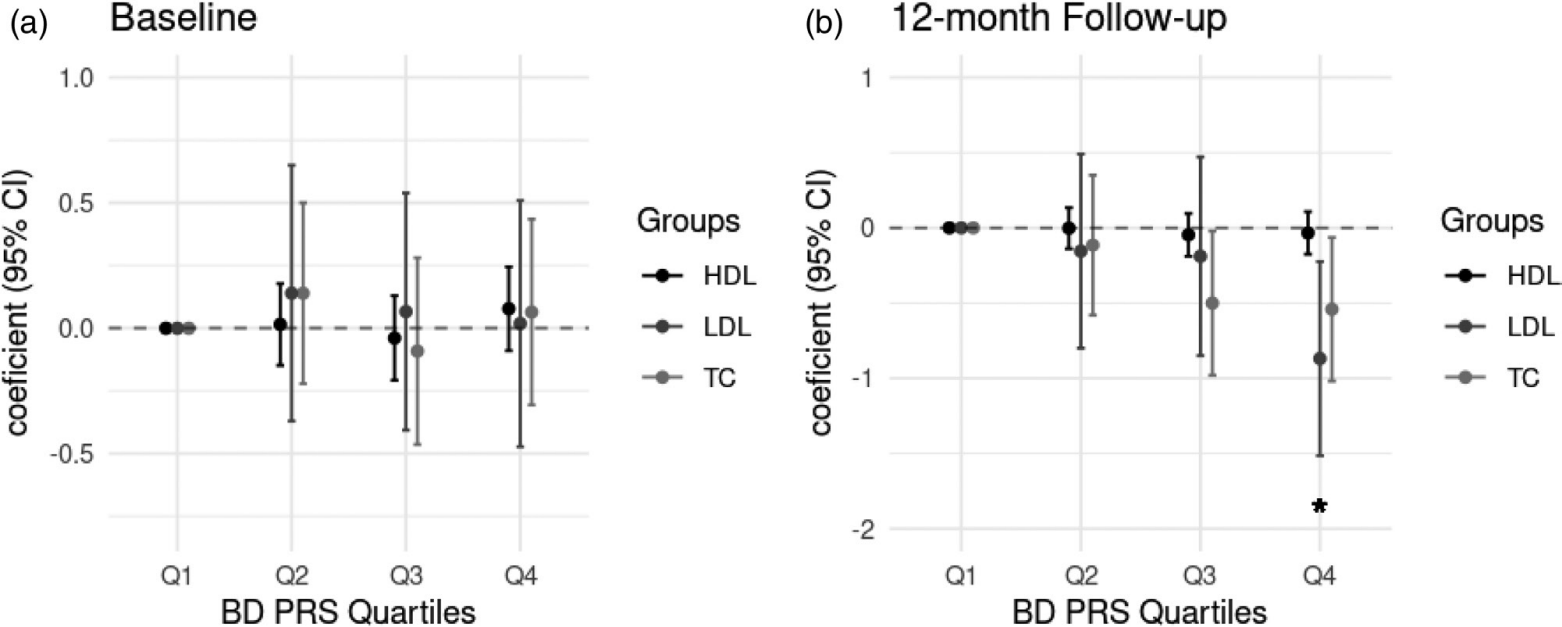
Comparison of baseline and 12-month follow-up cholesterol levels across bipolar disorder PRS quartile groups. (a) Cholesterol measures at baseline. (b) Cholesterol measures at 12-month follow-up. The plotted values represent the estimated changes relative to the lowest bipolar PRS quartile group with 95% confidence intervals. The 12-month follow-up models were adjusted for the corresponding baseline measure in addition to all previously described covariates.* In the current plot, *P* value survived FDR multiple testing correction. BD, bipolar disorder; CI, confidence interval; FDR, false discovery rate; HDL, high-density lipoprotein cholesterol; LDL, low-density lipoprotein cholesterol; PRS, polygenic risk scores; TC, total cholesterol.

To further explore the associations of bipolar disorder PRS with different cholesterol levels, we split the participants into four quartiles based on their bipolar disorder PRS and compared its influence between baseline and 12-month follow-up, where the 12-month follow-up models included the corresponding baseline measure as an additional covariate. At baseline, we found no association between bipolar disorder PRS and any of these cholesterol levels (Fig. 2a); however, at 12-month follow-up, the associations became apparent even if the baseline measure’s impact was controlled for (Fig. 2b). Participants in the top bipolar disorder PRS quartile group had 0.58 mmol/L lower LDL measures (*t* = −0.860; 95% confidence interval: −1.516 to −0.204; *P*_adj_ = 0.039) compared the lowest group. The overall model, including all covariates, could explain 25% of total variance in LDL measures at 12-month follow-up, while bipolar disorder PRS accounted for 6% of these variations. These findings further indicated bipolar disorder genetic risk potential impact on LDL treatment responses, and the relationship between LDL and bipolar disorder PRS was likely to account for the association of total cholesterol levels and bipolar disorder PRS.

## Discussion

In summary, we used multiple cardiovascular and psychiatric PRSs to investigate how different genetic risks can impact cardiovascular behavioral treatment responses in patients with SMI within primary care. Before treatment, most of these selected PRSs were predictive of the actual cardiovascular measures except for the LDL PRS. The interventions were effective in reducing total cholesterol and systolic blood pressure measures; however, the respective PRS became nonpredictive posttreatment. The genetic risks for HDL seemed to be predictive, independent of the treatment and baseline measures. The LDL measures were not associated with LDL PRS at both time points, but became negatively associated with bipolar disorder PRS after treatments, even though we found no significant interaction effects. These findings collectively suggest that PRS predictions could be influenced by treatment interventions.

The findings of the study may provide valuable insights into the potential heterogeneity within PRS predictions when applied to variable health measures. While an individual’s genetic risks remain stable, the changes in their prediction coefficients could reflect the treatment effects as coefficients represent the strength of associations between predictors and outcomes. For PRS coefficients that mostly remained stable or even increased after treatment, such as HDL, this may suggest that the overall treatment likely had little impact. In contrast, the coefficients attenuated toward the null for total cholesterol and systolic blood pressure measures at follow-up, on which the interventions had significant effects on. These findings underscore the importance of carefully considering the timing of PRS assessments for accurate risk prediction. Such findings also highlighted the need for more nuanced models that integrate both genetic and environmental factors to better predict individual treatment responses for patients with SMI.

For the participants’ psychiatric genetic impact on the treatment outcomes, we initially hypothesized that the participants with higher genetic risks might exhibit reduced treatment effectiveness; however, the results at the 12-month follow-up were in the opposite direction for bipolar disorder PRS. While it is well-documented that patients with bipolar disorder can have altered lipid profiles, previous findings have been inconsistent (Hiller *et al.*, 2023). Notably, studies that did find associations often reported a directional trend: depressive episodes tended to correlate with increased lipid levels, whereas manic episodes were more frequently associated with decreased lipid levels (Fusar-Poli *et al.*, 2021; Hiller *et al.*, 2023). Using total cholesterol, triglycerides, LDL, and HDL as biomarkers is inherently complex because of numerous potential confounders, including dietary intake, comorbid somatic conditions, and medication use (Katcher *et al.*, 2009). In this study, no prior association was observed between bipolar disorder PRS and LDL levels at baseline; however, a negative association with bipolar disorder PRS emerged after treatment. Again, such findings highlighted the instability of PRS associations.

Notably, bipolar disorder does not seem to share any genetic correlation with LDL according to the most recent and largest bipolar disorder GWAS to date (O’Connell *et al.*, 2025); however, a recent comprehensive MR study covering 179 lipid species and five psychiatric disorders identified that elevated levels of two sterol esters and eight phosphatidylcholines could be protective in bipolar disorder (van der Veen *et al.*, 2017; Yu *et al.*, 2025). The role of the phosphatidylcholines also appeared dualistic: while some phosphatidylcholines exhibit protective effects, others act as risk factors to promote the development of bipolar disorder. Our findings highlighted the complex relationship between bipolar disorder and LDL levels. The observed increase in association at follow-up could be partially explained by reductions in other confounding variables, as the participants indeed showed positive changes in some lifestyle and health measures; however, environmental risk factors continue to play a role, and future studies with increased sample size and specific splits on bipolar disorder subtypes may provide further clarity.

The study’s strengths were that we used longitudinal behavioral treatment data to indicate how cardiovascular PRS predictions within patients with SMI could change over time. We employed robust methodologies and utilized the latest GWAS for PRS calculations. The results presented here remained consistent and robust after accounting for the participants’ age, sex, group allocation, diagnoses, prescribed medications, smoking habits, alcohol consumption, and physical activity levels. In addition, we conducted comprehensive sensitivity analyses to validate the results, further reinforcing the credibility and generalizability of our findings. The findings we present can provide a solid foundation for future studies.

The study also had limitations that should be acknowledged. For instance, some of our health measures, such as medication use, were purely extracted from clinical records, which may have been incomplete or inconsistent. In addition, the participants’ compliance with treatment and medications may have influenced treatment effectiveness, but these could not be analyzed as adherence data were incomplete for most selected participants. The participants in the study had mixed diagnoses and lacked specific symptom measurements, thus could not allow us for more in-depth investigations. The included sample comprised a subset of clinical trial participants who consented to provide genetic data. As such, the cohort may reflect selection biases due to differential participation rates among those willing to undergo genetic testing. In addition, our samples were predominantly of European ancestry, which may have limited generalizability and transferability.

### Conclusion

In summary, our findings highlighted the instability of PRS for cardiovascular risk predictions within patients with SMI. Researchers should carefully consider the measurement time points as these could produce diverse results. Future studies with increased sample size and more precise symptom measurements on bipolar disorder subtypes may clarify the complex associations between bipolar disorder and cardiovascular traits.

## Acknowledgements

The views expressed are those of the authors and not necessarily those of the sponsor, the National Health Service (NHS), the NIHR, or the Department of Health and Social Care.

This paper summarizes independent research funded by the National Institute for Health Research (NIHR) under its Programme Grants for Applied Research scheme (grant reference number RP-PG-0609-10156).

Data are available upon reasonable request from the corresponding author.

### Conflicts of interest

There are no conflicts of interest.

## Supplementary Material


