# Supplementary material for: Polygenic risks and cardiovascular treatment effects in severe mental illness
Source: Psychiatr Genet. 2025 Oct 23;35(6):143–53. doi: 10.1097/YPG.0000000000000401 (PMC12588646; doi:10.1097/YPG.0000000000000401)
Supplement: Supplementary file 1 [file pg-35-143-s001.docx]

**Supplementary Materials**

**Polygenic Risks and Cardiovascular Treatment Effects in Severe Mental Illness**

Kai Yao^1^, Alexandra Burton^2^, Samira Heinkel^3^, David Osborn^3^, Nick Bass^1^, Andrew McQuillin^1^

**Table of Contents**

Supplementary Table 1. Adjusted Results of Multiple Regressions with corresponding Cardiovascular PRS on European Participants

Supplementary Table 2. Adjusted Results of Cardiovascular and Psychiatric PRS Interactions on European Participants

| **Supplementary Table 1. Adjusted Results of Multiple Regressions with corresponding Cardiovascular PRS on European Participants** | | | | | | | |
| --- | --- | --- | --- | --- | --- | --- | --- |
| **Variables** | **Coefficient** | **SE** | **t-statistics** | **CI (95%)** | | ***p*** | ***p.adj*** |
| **Total Cholesterol** |  |  |  | |  |  |  |
| Baseline | 0.211 | 0.065 | 3.272 | | 0.084, 0.339 | **0.001** | **0.007** |
| 12 Month | 0.154 | 0.096 | 1.603 | | -0.036, 0.345 | 0.111 | 0.195 |
| 12 Month BA | -0.024 | 0.093 | -0.253 | | -0.209, 0.161 | 0.801 | 0.841 |
| **HDL** |  |  |  | |  |  |  |
| Baseline | 0.173 | 0.025 | 6.847 | | 0.123, 0.223 | **<0.001** | **<0.001** |
| 12 Month | 0.213 | 0.031 | 6.956 | | 0.152, 0.274 | **<0.001** | **<0.001** |
| 12 Month BA | 0.064 | 0.026 | 2.409 | | 0.011, 0.116 | **0.026** | **0.049** |
| **LDL** |  |  |  | |  |  |  |
| Baseline | 0.084 | 0.081 | 1.036 | | -0.078, 0.246 | 0.303 | 0.398 |
| 12 Month | 0.098 | 0.127 | 0.768 | | -0.156, 0.352 | 0.445 | 0.550 |
| 12 Month BA | -0.032 | 0.126 | -0.256 | | -0.287, 0.222 | 0.800 | 0.841 |
| **Triglycerides** |  |  |  | |  |  |  |
| Baseline | 0.467 | 0.150 | 3.109 | | 0.169, 0.765 | **0.003** | **0.009** |
| 12 Month | 0.493 | 0.172 | 2.866 | | 0.15, 0.836 | **0.006** | **0.017** |
| 12 Month BA | 0.238 | 0.190 | 1.255 | | -0.143, 0.619 | 0.215 | 0.323 |
| **Systolic blood pressure** |  |  |  | |  |  |  |
| Baseline | 4.278 | 1.335 | 3.203 | | 1.639, 6.916 | **0.002** | **0.007** |
| 12 Month | 1.832 | 1.206 | 1.519 | | -0.556, 4.22 | 0.131 | 0.212 |
| 12 Month BA | 0.785 | 1.099 | 0.714 | | -1.391, 2.961 | 0.477 | 0.556 |
| **Diastolic blood pressure** |  |  |  | |  |  |  |
| Baseline | 1.783 | 0.843 | 2.116 | | 0.118, 3.448 | **0.036** | 0.076 |
| 12 Month | 1.633 | 0.878 | 1.861 | | -0.104, 3.37 | 0.065 | 0.124 |
| 12 Month BA | 0.896 | 0.785 | 1.141 | | -0.659, 2.451 | 0.256 | 0.359 |
| **Body Mass Index (BMI)** |  |  |  | |  |  |  |
| Baseline | 1.472 | 0.430 | 3.424 | | 0.622, 2.322 | **0.001** | **0.006** |
| 12 Month | 1.413 | 0.523 | 2.699 | | 0.377, 2.449 | **0.008** | **0.021** |
| 12 Month BA | -0.015 | 0.249 | -0.059 | | -0.508, 0.478 | 0.953 | 0.953 |
| *Notes.* SE=standard errors; CI=confidence interval; PRS=polygenic risk score.  Presented results are outputs from linear regressions on only European participants corresponding to Table 3. Participants’ sex, age, treatment allocation, diagnosis, smoking scores, alcohol scores, IPAQ scores and the first ten principal components from population stratification were added as covariates to all models. Each model was also adjusted for the corresponding medication recorded at baseline or 12-month assessment including antipsychotic, antidepressant, antihypertensive, mood stabilizers and other medications. 12 Month BA (baseline adjusted) models were further adjusted for the corresponding baseline measure.  P.adj are p values corrected using FDR method. P values in bold are smaller than the threshold at 0.05. | | | | | | | |

| **Supplementary Table 2. Adjusted Results of Cardiovascular and Psychiatric PRS Interactions on European Participants** | | | | | | |  |
| --- | --- | --- | --- | --- | --- | --- | --- |
| **Variables** | **Coefficient** | **SE** | **t-statistics** | **CI (95%)** | | ***p*** | ***p.adj*** |
| **Total Cholesterol** |  |  |  | |  |  |  |
| BD PRS | -0.254 | 0.092 | -2.758 | | -0.436, -0.072 | **0.007** | 0.094 |
| BD PRS Interaction | 0.125 | 0.099 | 1.264 | | -0.071, 0.32 | 0.209 | 0.730 |
| SCZ PRS | -0.199 | 0.101 | -1.969 | | -0.399, 0.001 | 0.051 | 0.359 |
| SCZ PRS Interaction | 0.013 | 0.102 | 0.131 | | -0.189, 0.216 | 0.896 | 0.969 |
| **HDL** |  |  |  | |  |  |  |
| BD PRS | 0.000 | 0.030 | 0.011 | | -0.058, 0.059 | 0.991 | 0.991 |
| BD PRS Interaction | -0.019 | 0.035 | -0.534 | | -0.088, 0.05 | 0.594 | 0.901 |
| SCZ PRS | -0.047 | 0.032 | -1.473 | | -0.111, 0.016 | 0.143 | 0.730 |
| SCZ PRS Interaction | -0.001 | 0.036 | -0.033 | | -0.073, 0.071 | 0.974 | 0.991 |
| **LDL** |  |  |  | |  |  |  |
| BD PRS | -0.325 | 0.108 | -3.017 | | -0.541, -0.109 | **0.004** | 0.094 |
| BD PRS Interaction | 0.111 | 0.110 | 1.016 | | -0.108, 0.331 | 0.314 | 0.894 |
| SCZ PRS | -0.185 | 0.130 | -1.417 | | -0.446, 0.076 | 0.162 | 0.730 |
| SCZ PRS Interaction | 0.016 | 0.124 | 0.126 | | -0.232, 0.263 | 0.900 | 0.969 |
| **Triglycerides** |  |  |  | |  |  |  |
| BD PRS | -0.181 | 0.139 | -1.309 | | -0.458, 0.095 | 0.195 | 0.730 |
| BD PRS Interaction | -0.096 | 0.161 | -0.592 | | -0.418, 0.227 | 0.556 | 0.901 |
| SCZ PRS | -0.153 | 0.153 | -1.002 | | -0.459, 0.152 | 0.320 | 0.894 |
| SCZ PRS Interaction | 0.074 | 0.172 | 0.431 | | -0.269, 0.417 | 0.668 | 0.901 |
| **Systolic blood pressure** |  |  |  | |  |  |  |
| BD PRS | 0.925 | 1.106 | 0.836 | | -1.265, 3.115 | 0.405 | 0.894 |
| BD PRS Interaction | -2.266 | 1.087 | -2.086 | | -4.417, -0.115 | 0.039 | 0.359 |
| SCZ PRS | -1.156 | 1.311 | -0.882 | | -3.752, 1.44 | 0.380 | 0.894 |
| SCZ PRS Interaction | -0.386 | 1.029 | -0.375 | | -2.422, 1.651 | 0.708 | 0.901 |
| **Diastolic blood pressure** |  |  |  | |  |  |  |
| BD PRS | -0.368 | 0.854 | -0.431 | | -2.059, 1.323 | 0.667 | 0.901 |
| BD PRS Interaction | -0.691 | 0.845 | -0.818 | | -2.365, 0.982 | 0.415 | 0.894 |
| SCZ PRS | -0.623 | 0.965 | -0.646 | | -2.533, 1.287 | 0.520 | 0.901 |
| SCZ PRS Interaction | -0.474 | 0.870 | -0.545 | | -2.196, 1.248 | 0.587 | 0.901 |
| **Body Mass Index (BMI)** |  |  |  | |  |  |  |
| BD PRS | 0.231 | 0.480 | 0.481 | | -0.719, 1.18 | 0.631 | 0.901 |
| BD PRS Interaction | -0.176 | 0.532 | -0.331 | | -1.229, 0.877 | 0.742 | 0.903 |
| SCZ PRS | -0.119 | 0.539 | -0.220 | | -1.186, 0.949 | 0.826 | 0.964 |
| SCZ PRS Interaction | -0.206 | 0.500 | -0.412 | | -1.197, 0.785 | 0.681 | 0.901 |
| *Notes.* SE=standard errors; CI=confidence interval; SCZ=schizophrenia; BD=bipolar disorder.  Presented results are outputs from linear regressions on only European participants corresponding to Table 4. P.adj are p values corrected using FDR method. P values in bold are smaller than 0.05. | | | | | | | |
